# Predisposing Factors to Medication Errors by Nurses and Prevention Strategies: A Scoping Review of Recent Literature

**DOI:** 10.3390/nursrep14030117

**Published:** 2024-06-26

**Authors:** Fábio Coelho, Luís Furtado, Natália Mendonça, Hélia Soares, Hugo Duarte, Cristina Costeira, Cátia Santos, Joana Pereira Sousa

**Affiliations:** 1 Department of Nursing, Mental Health, and Gerontology, School of Health, University of the Azores, 9700-042 Angra do Heroísmo, Portugal; luis.cr.furtado@uac.pt (L.F.); helia.m.soares@uac.pt (H.S.); 2 Flores Island Healthcare Unit, 9960-430 Flores Island, Portugal; natalia.lr.mendonca@azores.gov.pt; 3 Center for Innovative Care and Health Technology (ciTechCare), School of Health Sciences, Polytechnic of Leiria, 2411-090 Leiria, Portugalcristina.costeira@ipleiria.pt (C.C.); catia.santos@ipleiria.pt (C.S.); joana.sousa@ipleiria.pt (J.P.S.); 4 Health Sciences Research Unit: Nursing (UICISA: E), Nursing School of Coimbra (ESEnfC), 3004-011 Coimbra, Portugal

**Keywords:** interventions, nurses, medication errors, intensive care units

## Abstract

Medication errors have serious consequences and high costs for the patient and the system. The treatment process and the care required for critically ill patients are complex, and these patients are more vulnerable to errors and potential consequences. A scoping review using the JBI methodology was conducted across PubMed, CINAHL, and MEDLINE databases and reported by the PRISMA-ScR guidelines to explore strategies that can mitigate medication errors by nurses. The search strategy focused on references published between January 2012 and April 2023. Sixteen studies were included, and the results were organized into thematic areas. Medication errors by nurses are in the areas of preparation, administration, and documentation; organizational, system-related, procedural, personal, and knowledge and training factors are predisposing factors for errors; educational intervention, verification and safety methods, organizational changes, and error reporting are the strategic areas to mitigate medication error. The organization of the data could be different, as it depends on the reviewers’ experience. Knowledge of the factors that cause medication errors and interventions to mitigate them make it possible to outline strategies to minimize their occurrence and achieve health gains. The protocol preceding this review has been registered in the Open Science Framework and published.

## 1. Introduction

Patient safety has emerged as a critical concern in healthcare, with medication errors attracting significant attention due to their profound implications. Recent literature underscores the alarming frequency and severe consequences of these errors. For instance, studies estimate that medication errors affect approximately 1 in every 10 hospitalized patients, with nearly 7% of these errors resulting in fatalities [1]. These errors not only threaten patient lives but also impose exorbitant costs on healthcare systems, consuming valuable resources that could be directed towards improving patient care [1,2,3,4].

Nurses are integral to the healthcare system, particularly in managing patient safety during the medication process. This process is inherently complex and error-prone, encompassing various stages from the selection and storage of medications to their prescription, verification, preparation, administration, and monitoring [5,6]. Given their pivotal role, nurses are often the last line of defense against medication errors at the administration phase, which is noted for its high risk and correlation with adverse patient outcomes [7,8].

Intensive care units (ICUs) present unique challenges due to the critical nature of patients and the complexity of care required. Patients in ICUs typically undergo more frequent medication changes and receive a higher number of drugs, which significantly increases the risk of errors. Studies have shown that in ICUs, medication errors occur at a rate of approximately 1.7 per patient per day, with severe or fatal errors disproportionately affecting these units [9,10]. These errors often result from high-risk procedures, complex medication regimens, and the intense pace of work, which can overwhelm even the most diligent healthcare teams.

Given this backdrop, there is a pressing need to explore and understand the nuances of medication errors within the ICU setting. It is recognized that there are literature reviews that address issues related to error. However, other authors in this process have concluded that the evidence for effective interventions to reduce medication errors by nurses in adult ICUs is limited due to the inconsistency of the research design and methods [11]. Hence, the relevance of this review. This scoping review is designed to delve into the literature to unearth the multifactorial causes of medication errors and evaluate the effectiveness of interventions targeted at reducing these errors. The review will specifically focus on the nursing role within ICUs, identifying strategies that can enhance safety and minimize errors in medication administration [12,13].

The objectives of this review are to map out the contributory factors to medication errors, to assess the effectiveness of existing interventions, and to explore innovative strategies that can further mitigate these risks. The chosen methodological approach, a scoping review, is particularly suited for this purpose as it allows for a broad exploration of complex topics and facilitates the identification of key themes and gaps in the existing research.

The findings of this review are expected to contribute significantly to the body of knowledge on patient safety in ICUs. By identifying effective practices and areas needing improvement, this work aims to inform policy changes and guide the development of targeted training programs for nurses and other healthcare professionals. Ultimately, the insights garnered from this review will help shape interventions that are not only evidence-based but also contextually adapted to the demanding environment of intensive care, thereby enhancing patient safety and healthcare outcomes.

## 2. Methods

### 2.1. Research Question

To achieve the aim of this literature review study, a review question was formulated and organized following the PCC mnemonic (“population”, “concept”, and “context”) [14,15]. The study, therefore, sought to answer the following main question: Which interventions prevent medication errors by nurses at any stage of the medication management process in intensive care units?

Two supplementary questions further refine the scope of this review: (1) Which factors predispose nurses to medication errors in intensive care units? and (2) Which consequences and outcomes result from the occurrence of medication errors by nurses in intensive care units?

### 2.2. Study Design

The literature review followed the JBI methodology for scoping literature reviews [14,15]. This involved following a predetermined series of steps: (a) formulating the research question; (b) identifying relevant sources of evidence; (c) selecting sources of evidence for inclusion; (d) collecting/extracting data; and (e) grouping, summarizing, and reporting the findings. The results were presented following the Preferred Reporting Items for Systematic Reviews and Meta-Analyses Extension for Scoping Reviews (PRISMA-ScR) guidelines (Supplementary Materials Tables S1 and S2) [16,17]. The literature review protocol was registered on the Open Science Framework (https://doi.org/10.17605/OSF.IO/94KH3), and the protocol was also published [18].

### 2.3. Inclusion Criteria

In line with the JBI framework for scoping reviews of the literature [14], our review team established a comprehensive set of inclusion and exclusion criteria to guide the selection of studies. These criteria are essential for systematically assessing the records retrieved from various databases and platforms, ensuring the relevance and specificity of the included literature.

Participants/Population: This review focused on studies involving general care nurses, clinical nurse specialists, and advanced practice nurses, without restrictions regarding the length of professional practice or level of training. Excluded from this review were studies that primarily involved physicians, pharmacists, nursing assistants, or nursing students. However, for studies including multiple professional groups, data specifically pertaining to nurses were extracted if clearly identified in the original document. Studies lacking clear data on nurses or where such data could not be clarified by the authors were excluded.

Concept: The concept of intervention, often assumed as understood in various fields, lacks a thorough exploration in the literature, leading to a superficial understanding of its implications and effectiveness [19]. In this review, an intervention is defined as a specific set of activities implemented to operationalize an activity with known dimensions [20]. An intervention is considered effective if it yields the expected outcomes for the target population and context [21]. Medication errors are defined as failures in the medication management process that result in inappropriate medication use or have the potential to harm the patient [22]. Harm is described as temporary or permanent impairment of physical, emotional, or psychological functions or structures of the body, including pain requiring additional intervention [23]. Our review included studies addressing interventions designed to prevent medication errors specifically by nurses, encompassing any strategy aimed at mitigating such errors resulting from nursing actions

Context: This scoping review considered studies conducted in adult intensive care units. Studies referring to intermediate care units or pediatric and neonatal intensive care units were excluded. The exclusion of COVID-19-related studies is due to the heightened risk of medication errors during pandemics, which may stem from systemic failures, inadequate preparedness, increased staff stress, attrition, and emerging clinical complexities, necessitating distinct approaches to error mitigation [24].

Types of sources: The review included primary research employing quantitative, qualitative, and mixed methods that met the inclusion criteria. Additionally, reports and technical documents issued by recognized authorities such as government bodies, professional organizations, or scientific societies were considered for inclusion. Literature reviews, opinion texts, letters to the editor and editorials were excluded.

### 2.4. Search Strategy

The search strategy was meticulously structured in three stages following the JBI framework for scoping literature reviews [14], aiming to encompass a wide range of authoritative sources. This strategy targeted primary published studies, systematic reviews, technical documents, and reports from well-recognized databases, government bodies, professional organizations, scientific societies, and other entities with recognized expertise.

The period from January 2012 to April 2023 was selected to balance the scope of the review with the capacity and availability of reviewers. While this timeframe excludes earlier studies, it captures over a decade of relevant research, which we believe sufficiently represents recent trends and advances without significantly impacting the quality or conclusions of the review.

Documents and studies in English and Portuguese were included to accommodate the linguistic capabilities of the review team and the prevalence of these languages in the relevant research.

An initial search specifically targeted literature reviews on the topic but failed to yield results concerning interventions or factors that promote safety in medication practices by nurses in ICUs. This search was conducted on the Open Science Framework (OSF^®^, Center for Open Science, Charlottesville, VA, USA) using the terms “nursing”, “medication errors”, “intensive care unit”, and “review”, confirming the necessity and relevance of this comprehensive review.

A preliminary search was conducted in the PubMed electronic database to identify pertinent articles by analyzing text in titles, abstracts, and the index terms used. Terms from the natural vocabulary and descriptors identified during this preliminary phase were then utilized to refine the search strategy applied in subsequent database searches.

Originally, our protocol included plans to search the Excerpta Medica Database (Embase); however, access limitations prevented its inclusion in the final search strategy, which is noted as a limitation in the relevant section of this study. However, this strategy was expanded to include the Cumulative Index to Nursing and Allied Health Literature (CINAHL) Complete and the Medical Literature Analysis and Retrieval System Online (MEDLINE), both accessed via EBSCO (Supplementary Materials Table S3).

Searches were also conducted in the Open Access Scientific Repositories of Portugal (RCAAP) and Dart-Europe for unpublished studies, complemented by searches on websites of relevant professional associations, government bodies, and scientific societies to locate additional supporting documents.

In the final stage of the search, the reference lists of all retained documents were manually reviewed against the established inclusion and exclusion criteria to ensure comprehensiveness and relevance.

Table 1 shows the search strategy used in MEDLINE (via EBSCO), adapted to the other databases used, adjusting it to their specificities.

### 2.5. Study Selection

The obtained records were exported and imported into EndNote^®^ v.20.4 software (Clarivate Analytics, Philadelphia, PA, USA) for organization, analysis, and initial removal of duplicates. A random sample of 25 documents was obtained. It was subjected to a preliminary analysis by the reviewers, applying the defined eligibility criteria, followed by a meeting for discrepancy analysis and clarification of these criteria [14,25]. The compliance rate was over 75%.

The analysis, sorting, and selection process occurred in two distinct stages, carried out on a Rayyan^®^ platform (Qatar Computing Research Institute, Doha, Qatar). Initially, the records were imported into the Rayyan^®^ platform, where a secondary check for duplicates was performed. Subsequently, screening by title and abstract, based on predefined inclusion and exclusion criteria, was conducted by two independent, blinded reviewers. In a second stage, studies deemed eligible for full-text review were re-imported into Rayyan^®^, where they were once again assessed against the established eligibility criteria by two independent, blinded reviewers. Standardized reasons for exclusion were documented and reported. Any conflicts between reviewers were resolved through discussion or, if consensus could not be reached, the intervention of a third reviewer. Due to the nature and purpose of the review, the review team opted not to evaluate the methodological quality of the included studies [26]. The corresponding author was contacted whenever the information provided in the paper was insufficient or questionable. If the corresponding author did not respond and the sought-after information was crucial for the reliability of the extracted data, the study was excluded.

### 2.6. Data Extraction

The data were extracted utilizing a tool specifically designed for this purpose, developed within Microsoft Excel^®^ (Microsoft Corporation, Washington, DC, USA) and validated through testing on a random sample of 10 documents to ensure its clarity and efficacy in extracting relevant data for the study. No adjustments were deemed necessary following subsequent discussion meetings. For each included study, the following data points were extracted: year of publication, authors, journal name, title, country, study type, participants or number of documents included (depending on whether it was a primary study or a review study), objectives, as well as elements pertinent to and supporting the concept of interest and addressing the established research questions.

Data extraction was a collaborative effort, independently and blindly conducted by two reviewers. To enhance the efficiency of data extraction and familiarization with the extraction tool, a random sample of three articles was selected for preliminary extraction by all authors. This was followed by an analysis and collaborative meeting to standardize criteria, ensuring that reviewers extracted relevant data from the retained documents in accordance with the review’s objectives.

### 2.7. Data Synthesis and Reporting

A third reviewer consolidated the data independently extracted by other reviewers into a unified document, presented descriptively with supporting tables. Textual data underwent content analysis using an inductive approach, leading to the development of a coding structure for classifying, categorizing, and thematically grouping the data based on similarities and thematic connections. This structured analysis facilitated a clear understanding of the emerging themes relevant to the research questions.

## 3. Results

### 3.1. Characterisation of the Reported Studies

**Figure 1:**
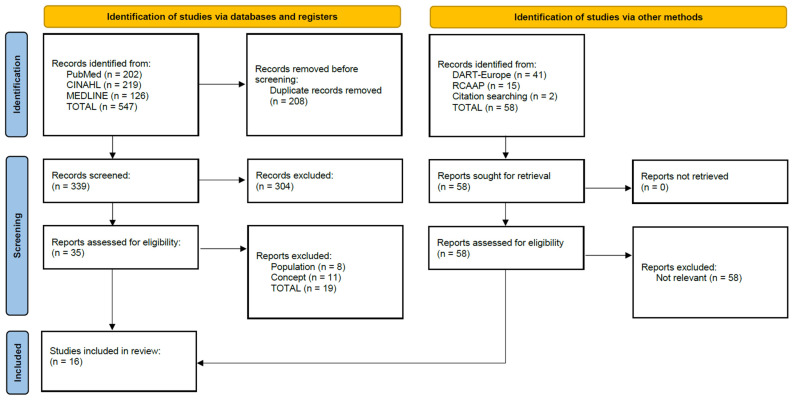
Flowchart representing the process of identifying, screening, and selecting the references included in the scoping review.

The database search identified 547 references. After screening and selecting the records, it was decided to include 16 articles in the review. The flowchart in Figure 1 illustrates the total number of identified records, along with the included and excluded reports, indicating the reasons for exclusion and the documents included after manual examination of the reference lists.

The publication trend showed a notable peak in 2020 with four studies, reflecting heightened interest in the topic. This was followed by 2017, with three studies, and 2012 and 2018, with two publications each. The remaining years saw a single publication each, suggesting a steady but less pronounced interest over time.

The studies were carried out in different countries, with three publications in the United States of America, three in Spain, two in Australia, and the others in different countries worldwide (Switzerland, Iran, Brazil, Malaysia, Italy, Egypt, China, and Norway). Methodologically, the studies encompassed a diverse range of designs including observational, time series, continuous improvement projects, exploratory, descriptive, cross-sectional, prospective controlled, mixed methods, narrative, and longitudinal studies (Supplementary Materials Table S4)

**Figure 2:**
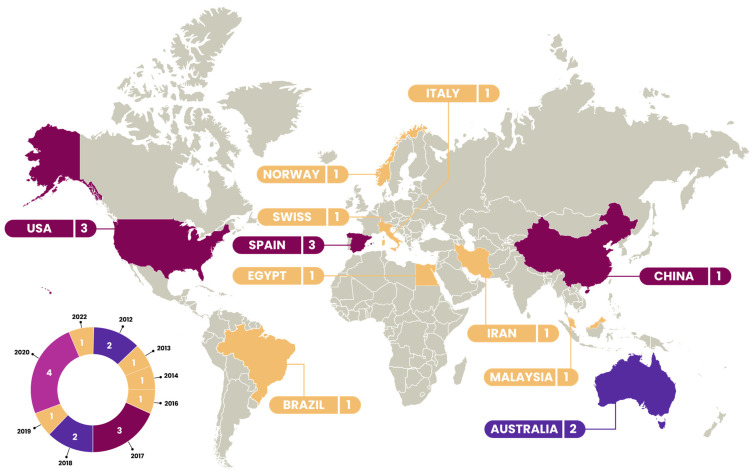
Geographical and temporal distribution of the studies included in the scoping review.

Figure 2 shows the geographical and temporal distribution of the studies included in this scoping review.

The data extracted from the included studies were analyzed according to four main dimensions: types of medication errors, predisposing factors for these errors, their consequences, and interventions to minimize them. These dimensions were further organized into categories and subcategories (Table 2), which are detailed in the following sections.

### 3.2. Types of Errors

In the analysis of medication errors, three primary categories were identified: preparation error, administration error, and documentation error.

Errors in medication preparation included incorrect labeling [27], expired infusions [27,28], and dilution errors [29,30].

The medication administration errors identified were: error of omission, in which the medication is prescribed but not administered [28,29,31,32]; non-interruption of medication, in which the medication is administered although the order to interrupt is given [31]; incorrect speed/time of administration [27,29,30,31,35,36]; drug incompatibility [27,29,35]; unauthorized/prescribed administration [32]; duplicate administration of the drug [39]; non-aseptic technique, such as not frictioning the connection door [28,36]; interruptions during administration [40]; errors in the handling of the pharmaceutical form to enable administration, such as crushing pills [30,35]; wrong frequency [29,31]; incorrect dosage/missed doses [27,29,31,33,34,35]; incorrect route of administration [29,31]; scheduling error [29,33,38]; and wrong medication [31,37].

The documentation errors associated with nurses’ actions identified were transcription errors between the prescription and the administration plan, namely the use of abbreviations, lack of dose, route of administration and schedule [29,30,31]; and lack of validation/incorrect recording of medication administration [27,28].

### 3.3. Factors Predisposing to Error

Five categories were identified regarding the factors that potentially influence nurses’ occurrence of medication errors: organizational factors, knowledge and training, system-related factors, personal factors, and procedure-related factors.

#### 3.3.1. Organizational Factors

Workload factors such as work overload [27,29,34,37,40,41], particularly during night shifts [37,38,40], and a reduced number of nursing staff [38] have been identified as conditions linked to the occurrence of medication errors. A lower staffing level not only complicates the implementation of improvement interventions but also degrades the standard of care. This necessitates adaptations to the workload, forcing priorities to be altered and some interventions to be favored over others [11].

Additionally, information overload [32] and time constraints or pressure [27,32,33] are significant factors that contribute to medication administration errors [32]. These conditions also pose challenges to the effective implementation of strategies aimed at improving the medication administration process [27].

#### 3.3.2. Knowledge and Training

Medication errors are often linked to a lack of knowledge in adhering to the five “rights” of medication [27,33]. A misperception of error risk highlights a deficit in understanding the medication-related factors that can lead to mistakes [32]. In particular, nurses’ limited familiarity with the medications most commonly used in ICU settings contributes to a higher rate of errors [29].

Additionally, a relationship has been observed between the length of professional experience and the frequency of medication errors. Findings indicate that nurses with more years of experience tend to demonstrate safer practices and are less prone to errors, compared to those with less experience or recent graduation [34,37]. Surprisingly, nurses holding a university degree were found more likely to engage in incorrect medication management behaviors than those without a degree [34].

#### 3.3.3. System-Related Factors

Issues such as an inadequate physical environment [33] and various system deficiencies in tasks and processes contribute to medication errors [41]. Furthermore, ambiguous documentation rules result in varying interpretations by nurses, leading to inconsistent information in the administration plans [31]. The lack of systematic feedback on potential error-inducing factors leaves professionals unaware of crucial points for improvement [27,32]. Effective feedback mechanisms, like those derived from a well-implemented reporting system, are essential for identifying and resolving system flaws [27].

#### 3.3.4. Personal Factors

Personal factors significantly affect the reliability of the drug safety process. Fatigue [33], distractions, and interruptions during critical tasks such as transcription or administration can lead to errors [28,29,40]. Poor interpersonal relationships within the work environment also contribute to these issues [36]. Minimizing interruptions, while considering the relevance of the interrupted information, is crucial for reducing errors [28].

#### 3.3.5. Factors Related to Procedures

Certain nursing procedures, like the manual preparation of infusions, often result in concentrations that deviate from those intended [36]. Transcription errors due to ambiguous instructions, unclear dosages, or omitted administration details also lead to significant mistakes [30,31].

### 3.4. Consequences of Error

Medication errors range from causing no harm to resulting in patient incapacity or even death [28,33,34]. These errors not only harm patients but also incur substantial costs due to increased adverse events and prolonged hospital stays [32,41]. Even non-critical errors can escalate care levels, necessitating further tests or specialist referrals, potentially leading to a permanent reduction in patient functional capacity and extended hospitalization [28,30,32].

### 3.5. Error Mitigation Interventions

Despite initial plans, the literature review did not categorize interventions by error type due to the studies’ organizational limitations. Instead, four broad categories of interventions were recognized: educational, verification and safety methods, organizational and functional modifications, and error reporting.

#### 3.5.1. Educational Intervention

The use of posters in strategic places where nurses work [34,36,39,42] is an educational and informative strategy, and nurses are encouraged to read and consult the posters during the preparation and administration of intravenous drugs [42].

The posters can be constructed in table form, with each line containing information on the preparation and administration of each commonly used medication [42], but also in the format of quick guides, step-by-step instructions explaining the procedures [39].

The distribution of pamphlets or information leaflets [32,34,42] is essential to educate nurses about the importance of checking the expiry dates of medicines, disinfecting hands before preparing medicines and inspecting clarity solutions [42]. The memory aids were developed in the size of a marker, containing a preparation and administration guide for medicines and calculation tips to serve as a quick reference, and distributed to all nurses [36].

Training and awareness sessions [27,32,34] are an intervention used to promote medication safety. They are usually presented by a qualified professional and held at a time set aside for staff on duty, where specific medicines, topics related to the administration of medicines, and available resources on medicines are covered [27]. Feedback sessions [27,34], in turn, allow for a review of reported medication errors and possible solutions [28], as well as raising awareness of errors and clinical risk management [34].

Discussion groups [27,39] about medication process safety can be held at staff meetings with the involvement of the multidisciplinary team, and between 5 and 10 min can be set aside for this purpose [27]. In addition, discussions can be considered during the daily staff sessions at the beginning of each shift [39].

Online resources [27,32] should be made available as a training and information-gathering strategy, and dissemination and training on their use should be considered, whether for checking drug compatibility [27] or for obtaining any other information about drugs [32].

The use of medication error prevention measures also includes training in simulated practice [28,34,39], such as the preparation and administration of intravenous medication to a mannequin patient [28].

Educational videos [36] can be used as a complement to other educational intervention measures, such as memory aids and the use of PowerPoint presentations, to enhance training moments [36]. Educational videos to minimize medication error can contain a practical demonstration of the drug preparation and administration processes, information about general guidelines on reconstitution/dilution, drug compatibilities, administration rates, aseptic technique, and other relevant practices [36].

#### 3.5.2. Verification and Security Methods

To streamline the medication process and reduce transcription errors, multifunctional forms have been developed [31]. These forms combine the prescription note and administration plan into a single document, which includes comprehensive details for each medication such as prescription, preparation, administration, and a section to note any discontinued medications. This integration facilitates clear communication and reduces errors related to medication continuity [31].

Standardized operating procedures serve as effective medication error prevention measures [34,35,39]. Protocols provide detailed instructions on the correct use of medications and are supported by the best available scientific evidence, ensuring reliability and promoting consistent practices across healthcare settings [35].

Checklists are used to verify each step in the medication administration process, enhancing safety and minimizing errors [32]. This strategy is complemented by routine checks of laboratory values before administering medications, especially intravenous ones, and by monitoring patients’ vital signs before and after administration [32,34]. Nurses are also encouraged to contact prescribers if there are any doubts regarding the medication orders [32,38].

Minimizing disruptions during medication preparation and administration is crucial for reducing errors [28,38]. It is important to evaluate the necessity of interruptions, ensuring that only essential communications occur during critical tasks.

Strategies like double-checking preparations by two nurses—one preparing and another verifying—help reinforce this practice and maintain workflow coordination [38]. Implementing a double-check system, where one nurse prepares all medications for a shift and another independently verifies them, significantly reduces the risk of errors. This practice not only ensures accuracy but also builds a safety culture focused on collaboration and meticulous verification [38].

#### 3.5.3. Organizational and Functional Changes

Using strategies that involve modifying organizational and functional aspects, such as using a different color system, designs, and labels that make it possible to identify the different medication boxes, results in efficient measures to reduce errors [37]. In addition, separating similarly labeled medicines in different locations is also an effective measure for reducing medication errors [37]. The involvement of new technologies in the care process has been pointed out as a possible error prevention measure, for example, the use of medication administration technologies, such as the use of bar-coded medication and the use of an electronic documentation system rather than paper documentation systems [37].

#### 3.5.4. Error Notification

A robust incident reporting system is vital for evaluating and improving medication safety processes [27,34,41]. Error reporting is necessary for improving the service and reducing errors [34]. In addition, medication errors should be reported to the physician and pharmacy service, as well as to team members and supervisors, so that measures can be taken to prevent complications [29].

Promoting a culture that views error reporting as a tool for improvement rather than punishment is essential for identifying systemic issues and enhancing overall safety [27]. To help the error reporting process, a computer is needed in each patient unit; time is needed for reporting; a more efficient reporting method is created; an anonymous and depersonalized system is prioritized; greater awareness among nurses that error reporting is intended to identify points for improvement and system errors and not personal faults; greater awareness of the importance of the results of reporting; more training on the incident reporting system; and ensuring that reporting is not a punishment, but an improvement strategy [27].

The reports of medication-related events that emerge from the reporting of errors can be relevant tools to use in feedback sessions to mitigate future events. They provide a solid source of information about possible gaps in the safety of the medication management process and can offer opportunities for improving the system [41].

## 4. Discussion

The findings of this review were categorized into four key dimensions: types of errors, predisposing factors for errors, consequences of errors, and interventions to mitigate medication errors. These dimensions provided comprehensive answers to the initial research questions and objectives of this literature review.

While all included studies mentioned types of medication errors, only six explicitly addressed the consequences of these errors [28,30,32,33,34,41]. Medication management is inherently complex and error-prone, particularly during the prescription, verification, preparation, administration, and monitoring phases [6]. Notably, the majority of impactful medication errors occur during the preparation and administration phases—key areas of nursing practice [3,43]. It is crucial that nurses engage in thorough analyses of errors in these phases to enhance medication safety.

Medication errors stem from a blend of individual, organizational, task-related, work, and team factors [44]. These errors are often procedural, arising from activities such as the transcription process [30,31] and manual medication preparation [36]. The predominant predisposing factors identified include work overload and a lack of knowledge, which are intricately linked with broader health service management issues [3].

While personal factors such as knowledge, attitude, and behavior are critical, institutional factors such as staffing levels, workload, shift patterns, and physical working conditions are major contributors to medication errors [45,46,47]. This aligns with findings from this review, emphasizing that systemic issues are significant drivers of errors.

The relationship between a nurse’s education level and medication errors is complex. Higher education levels have been associated with both an increase and a decrease in medication errors [34,48,49], suggesting variability in how education impacts error rates. However, more consistent findings indicate that increased professional experience correlates with fewer errors [34,37]. This highlights the value of practical experience in enhancing medication safety, where practical experience seems to play a more significant role in reducing medication errors than formal education alone.

Interventions identified in this review include educational programs, training, double-check systems, procedure standardization, and the use of advanced technology such as barcode medication labeling [47,50]. Creating a culture where error reporting is encouraged and non-punitive can lead to significant improvements in safety practices [3,45,51].

Efforts to mitigate medication errors should focus on alleviating known risk factors as much as possible. Strategies should include educational enhancements, system corrections, and fostering a reporting culture that encourages the communication of all error types, not just the most severe [51]. The introduction of transformational leadership could further improve supervision and cultivate a safety-oriented culture [52].

Developing strategies based on error reports can help identify the causes of errors and improve medication safety. The challenge remains, however, to overcome the culture of underreporting due to fear, guilt, and the stigma of punishment [2,11,47,53,54].

The results from the included studies support a comprehensive approach to improving medication safety, focusing on education, communication, and leadership strategies. Nursing managers are tasked with identifying system flaws and developing plans that minimize errors and optimize outcomes in intensive care settings.

### 4.1. Implications for Nursing Practice

Understanding the factors that contribute to medication errors underscores the need for multifaceted improvement interventions at various levels, including system design, organizational structure, clinical practices, and working conditions. The high-stakes environment of an ICU demands targeted attention to effectively mitigate medication errors made by nurses.

The results obtained from this review study could influence the implementation of strategies that enhance safety and reduce medication errors in intensive care settings. The diversity of the studies reviewed provides a robust foundation for managers and nursing professionals to enhance the efficiency of care delivery, ensuring it is safe, responsible, and ethically sound. Additionally, these insights support organizations in improving working conditions to achieve excellence in patient care.

While the optimal interventions for error prevention are not infallible, managers must strategically navigate resource constraints and prioritize interventions that are collectively beneficial and supported by strong evidence, tailored to specific contexts.

The implementation of robust error reporting tools is crucial. These tools assist nursing managers in identifying systemic weaknesses, facilitating the development of comprehensive personal and professional growth plans for their teams. It is essential for healthcare professionals to recognize the importance of reporting adverse events, including medication errors. Such reporting is critical to fostering a safety culture that prioritizes minimizing harm, learning from incidents, and continually improving care quality.

### 4.2. Limitations

This literature review acknowledges several limitations that warrant consideration and analysis. One notable limitation is the restriction on the publication timeframe of the included studies, spanning from January 2012 to April 2023. This range was chosen because it encompassed a period with a high concentration of relevant articles, reflecting more contemporary professional contexts and practices. While this focus on recent literature helps ensure the applicability of findings to current settings, it also means that valuable insights from earlier studies may have been omitted, potentially limiting the breadth of evidence reviewed.

Additionally, the methodological quality of the included studies was not assessed, a decision not typically required for this type of review but one that could influence the depth and reliability of the findings. This choice was made to avoid excluding potentially relevant studies that, despite methodological limitations, could offer valuable insights into the topic. This approach was intended to maximize the contribution of each study to the comprehensive mapping of factors and interventions related to medication errors by nurses in ICUs.

Another limitation is the exclusive focus on studies conducted in ICUs. This specificity may overlook relevant data from other healthcare settings, such as medical-surgical units, where different mitigation strategies might have been identified. Although these findings could be applicable to the ICU context, their transferability is not guaranteed.

The categorization of data into defined categories and subcategories was based on the reviewers’ interpretations and expertise, which, although validated by experts, could be viewed as subjective. Different researchers could have categorized and interpreted the data differently, which is a recognized limitation of this review.

From the point of view of the conclusions to be drawn, it is recognized that it is impossible to generalize widely from a geographical point of view. For example, there is an urgent need to promote a multicenter, transnational study, including several countries from different regions, to obtain a broad characterization of the problem.

Despite these limitations, the findings of this review are considered valuable within the context of the study type and its objectives. The limitations do not detract from the study’s contributions to understanding and addressing medication errors in intensive care settings.

## 5. Conclusions

We believe that the results of this review study have helped to overcome the limitations identified in previous studies on the same topic, particularly in terms of the inconsistency of the research design and methods, fundamentally due to the methodological choice made, i.e., the use of a scoping review, making it possible to scan the evidence published on the topic without the restrictions imposed by other methodological approaches in terms of the design of primary studies.

This review has elucidated a variety of errors stemming from nursing practices and identified a comprehensive set of factors and interventions aimed at minimizing medication errors in ICUs. These include organizational aspects, knowledge and training needs, system-related issues, procedural dynamics, and personal factors, all of which are consistent with the existing literature. The interventions identified, such as educational programs, safety checks, organizational modifications, and robust error reporting mechanisms, align well with the findings of this review.

Despite existing programs designed to reduce medication errors, the persistence of these errors highlights an ongoing challenge. This underlines the necessity for continued research in this vital area of nursing practice. Future studies should focus not only on testing and refining strategies to minimize medication errors but also on exploring the causal relationships between different types of errors and their impacts.

The significance of continuous monitoring, regular audits, and the promotion of non-punitive error reporting cannot be underestimated. These practices are essential for analyzing contributory factors to errors and for improving the overall conditions within healthcare systems.

There is a critical need to enhance health service quality through strategic management changes and appropriate organizational reforms. Moderating the nursing workload, reducing non-essential nursing activities, boosting professional motivation, and enhancing nurses’ knowledge and working conditions are imperative.

Understanding the factors that contribute to medication errors in intensive care settings is crucial for developing effective mitigation strategies. This knowledge not only aids in providing excellent healthcare but also helps in fostering a robust culture of safety.

## Figures and Tables

**Table 1:** Search strategy conducted in the MEDLINE (via EBSCO).

Search No.	Search Terms and Expressions	Results
S1	MH “Nurses” OR TI nurs* OR AB nurs*	549,244
S2	MH “Physicians” OR MH “Students+” OR MH “Nursing Assistants” OR TI physician* OR AB physician* OR TI student* OR AB student* OR TI “nursing assistant*” OR AB “nursing assistant*” OR TI “nursing student*” OR AB “nursing student*” OR TI “medical student*” OR AB “medical student*” OR TI undergraduate OR AB undergraduate OR TI “nursing aide*” OR AB “nursing aide*” OR TI “nursing assistant*” OR AB “nursing assistant*”	943,938
S3	S1 NOT S2	454,611
S4	MH “Treatment Errors” OR MH “Medication Errors” OR MH “Health Care Errors” OR TI “nursing error*” OR AB “nursing error*” OR TI “medical error*” OR AB “medical error*” OR TI “medication error*” OR AB “medication error*” OR TI “medication administration error*” OR AB “medication administration error*” OR TI “medication preparation error*” OR AB “medication preparation error*”	2763
S5	MH “Intensive Care Units” OR MH “Respiratory Care Units” OR MH “Coronary Care Units” OR TI “intensive medical care*” OR AB “intensive medical care*” OR TI “intensive care*” OR AB “intensive care*” OR TI ICU OR AB ICU OR TI “care, intensive” OR AB “care, intensive” OR TI “intensive care unit*” OR AB “intensive care unit*” OR TI “intensive care medicine” OR AB “intensive care medicine” OR TI “respiratory care unit*” OR AB “respiratory care unit*” OR TI “coronary care unit*” OR AB “coronary care unit*”	245,845
S6	MH “Intensive Care Units, Pediatric” OR MH “Intensive Care Units, Neonatal” OR TI “intensive care units, pediatric” OR AB “intensive care units, pediatric” OR TI “intensive care units, neonatal” OR AB “intensive care units, neonatal”	26,934
S7	S5 NOT S6	218,911
S8	S3 AND S4 AND S7	198
S9	S3 AND S4 AND S7 from 2012–2023	126

**Table 2:** Dimensions, categories, and subcategories mapped, and respective studies in which they were identified.

Dimension	Category	Subcategory	Study
Types of errors	Preparation errors	Incorrect labelling	[27]
		Expired infusion	[27,28]
		Dilution errors	[29,30]
	Administration errors	Omission	[28,29,31,32]
		Non-interruption	[31]
		Wrong frequency	[29,31]
		Incorrect dosage	[27,29,31,33,34,35]
		Incorrect speed of administration	[27,29,30,31,35,36]
		Incorrect route	[29,31]
		Incorrect medication	[31,37]
		Drug incompatibility	[27,29,35]
		Timetable error	[29,33,38]
		Non-authorised/prescribed administration	[32]
		Double administration	[39]
		Non-aseptic technique	[28,36]
		Interruption during administration	[40]
		Inadequate handling of the therapeutic form	[30,35]
	Documentation errors	Transcription failures	[29,30,31]
		Lack of validation	[27,28]
Factors predisposing to error	Organisational constraints	Work overload	[27,29,34,37,40,41]
		Night time	[37,38,40]
		Low number of nurses	[38]
		Information overload	[32]
		Time constraints/Time pressure	[27,32,33]
	Knowledge and training	Lack of knowledge	[27,29,32,33,37]
		Level of training	[34]
		Length of professional experience	[34,37]
	System-related factors	Inadequate physical environment	[33,38,41]
		Lack of rules regulating documentation	[31]
		Lack of system feedback	[27,32]
	Personal factors	Fatigue	[33]
		Distraction	[28,29,40]
		Poor relations with the work environment	[29]
	Procedure-related factors	Manual preparation of drugs	[36]
		Transcription faults	[30,31]
Error mitigation interventions	Educational intervention	Posting of posters	[34,36,39,42]
		Distribution of pamphlets/information leaflets	[32,34,42]
		Training/sensitization sessions	[27,32,34]
		Feedback sessions	[27]
		Discussion groups	[27,39]
		Online drug safety resources	[27,32]
		Frequent training/simulation training/practical training	[28,34,39]
		Educational videos	[36]
		Memory aids	[36]
		PowerPoint presentations	[36]
	Verification and safety methods	Creation of multifunctional forms	[31]
		Use of drug administration checklists	[32]
		Checking laboratory values before administration	[32]
		Contacting the prescriber if in doubt	[32]
		Monitoring vital signs before and after drug administration	[34]
		Reducing the frequency of interruptions	[28]
		Use of protocols	[34,35,39]
		Double-checking drug preparation	[38]
	Organisational and functional changes	Different colors, designs, and labels to identify different drug recipients	[37]
		Storing medicines with similar labels in different places	[37]
		Use of drug administration	[37]
	Error reporting	Implementation of an error reporting system	[27,34]
		Reporting of medication-related events	[41]
		Error communication	[29]

## Data Availability

Not applicable.

## Supplementary Materials

The following supporting information can be downloaded at: https://www.mdpi.com/article/10.3390/nursrep14030117/s1, Table S1: Preferred Reporting Items for Systematic Reviews and Meta-Analyses for Scoping Reviews (PRISMA-ScR) Checklist; Table S2: Abstract reporting checklist required from the PRISMA-ScR; Table S3: Search strategy conducted in the PubMed, CINAHL (via EBSCO) and MEDLINE (via EBSCO) databases, on 8 March 2023; Table S4: Overview of the characteristics of the studies included in the scoping review.

## Public Involvement Statement

No public involvement in any aspect of this research.

## Guidelines and Standards Statement

This manuscript was drafted against the PRISMA-ScR (Tricco AC, Lillie E, Zarin W, O’Brien KK, Colquhoun H, Levac D, Moher D, Peters MDJ, Horsley T, Weeks L, Hempel S, Akl EA, Chang C, McGowan J, Stewart L, Hartling L, Aldcroft A, Wilson MG, Garritty C, Lewin S, Godfrey CM, Macdonald MT, Langlois EV, Soares-Weiser K, Moriarty J, Clifford T, Tunçalp Ö, Straus SE. PRISMA Extension for Scoping Reviews (PRISMA-ScR): Checklist and Explanation. Ann Intern Med. 2018. PMID: 30178033) for scoping review research.
