# Supplementary material for: Predisposing Factors to Medication Errors by Nurses and Prevention Strategies: A Scoping Review of Recent Literature
Source: Nurs Rep. 2024 Jun 26;14(3):1553–69. doi: 10.3390/nursrep14030117 (PMC11270417; doi:10.3390/nursrep14030117)
Supplement: Supplementary file 1 [file nursrep-14-00117-s001.zip › Table_S1.pdf]

**Table S1.** Preferred Reporting Items for Systematic Reviews and Meta-Analyses for Scoping Reviews (PRISMA-ScR) Checklist [15,16]

| SECTION AND TOPIC | ITEM # | PRISMA-SCR CHECKLIST ITEM | Location where item is reported |
|---|---|---|---|
| **Title** | | | |
| Title | 1 | Identify the report as a scoping review. | Title |
| **Abstract** | | | |
| Structured summary | 2 | See the PRISMA 2020 for Abstracts checklist. | Supplementary table 2 |
| **Introduction** | | | |
| Rationale | 3 | Describe the rationale for the review in the context of what is already known. Explain why the review questions/objectives lend themselves to a scoping review approach. | Paragraphs 4, 5 and 6 |
| Objectives | 4 | Provide an explicit statement of the questions and objectives being addressed with reference to their key elements (e.g., population or participants, concepts, and context) or other relevant key elements used to conceptualize the review questions and/or objectives. | Paragraphs 5, 6 and 11-14 |
| **Methods** | | | |
| Protocol and registration | 5 | a) Provide registration information for the review, including register name and registration number, or state that the review was not registered.<br>b) Indicate where the review protocol can be accessed, or state that a protocol was not prepared.<br>c) Describe and explain any amendments to information provided at registration or in the protocol. | Abstract and paragraphs 9 and 20 |
| Eligibility criteria | 6 | Specify characteristics of the sources of evidence used as eligibility criteria (e.g., years considered, language, and publication status), and provide a rationale. | Paragraphs 16 and 17 |
| Information sources | 7 | Describe all information sources in the search (e.g., databases with dates of coverage and contact with authors to identify additional sources), as well as the date the most recent search was executed. | Paragraphs 16 and 18-21 |
| Search | 8 | Present the full search strategies for all databases, registers and websites, including any filters and limits used. | Supplementary table 3 |
| Selection of sources of evidence† | 9 | Specify the methods used to decide whether a study met the inclusion criteria of the review, including how many reviewers screened each record and each report retrieved, whether they worked independently, and if applicable, details of automation tools used in the process. | Paragraphs 24-28 |
| Data charting process | 10 | a) List and define all outcomes for which data were sought. Specify whether all results that were compatible with each outcome domain in each study were sought (e.g. for all measures, time points, analyses), and if not, the methods used to decide which results to collect.<br>b) List and define all other variables for which data were sought (e.g. participant and intervention characteristics, funding sources). Describe any assumptions made about any missing or unclear information. | Paragraphs 15, 16 and 26 |
| Data items | 11 | List and define all variables for which data were sought and any assumptions and simplifications made. | NA |

| | | | |
|---|---|---|---|
| Critical appraisal of individual sources of evidence§ | 12 | If done, provide a rationale for conducting a critical appraisal of included sources of evidence; describe the methods used and how this information was used in any data synthesis (if appropriate). | NA |
| Synthesis of results | 13 | Describe the methods of handling and summarizing the data that were charted. | Paragraphs 26 and 27 |
| **Results** | | | |
| Selection of sources of evidence | 14 | a) Describe the results of the search and selection process, from the number of records identified in the search to the number of studies included in the review, ideally using a flow diagram.<br>b) Cite studies that might appear to meet the inclusion criteria, but which were excluded, and explain why they were excluded. | Figure 1.<br>NA |
| Characteristics of sources of evidence | 15 | For each source of evidence, present characteristics for which data were charted and provide the citations. | Figure 2. Supplementary tables 4. Paragraphs 30-33. |
| Critical appraisal within sources of evidence | 16 | If done, present data on critical appraisal of included sources of evidence (see item 12). | NA |
| Results of individual sources of evidence | 17 | For each included source of evidence, present the relevant data that were charted that relate to the review questions and objectives. | Table 2. Supplementary tables 4. Paragraphs 34-64. |
| Synthesis of results | 18 | Summarize and/or present the charting results as they relate to the review questions and objectives. | Figure 2. Supplementary tables 4. Paragraphs 34-64. |
| **Discussion** | | | |
| Summary of evidence | 19 | Summarize the main results (including an overview of concepts, themes, and types of evidence available), link to the review questions and objectives, and consider the relevance to key groups. | Paragraphs 65-73. |
| Limitations | 20 | Paragraph | Paragraphs 78-82. |
| Conclusions | 21 | Provide a general interpretation of the results with respect to the review questions and objectives, as well as potential implications and/or next steps. | Paragraphs 74-77. |
| **Other information** | | | |
| Funding | 22 | Declare any competing interests of review authors | Paragraph 96. |

NA: not applicable.
