# Supplementary material for: Predisposing Factors to Medication Errors by Nurses and Prevention Strategies: A Scoping Review of Recent Literature
Source: Nurs Rep. 2024 Jun 26;14(3):1553–69. doi: 10.3390/nursrep14030117 (PMC11270417; doi:10.3390/nursrep14030117)
Supplement: Supplementary file 1 [file nursrep-14-00117-s001.zip › Table_S2.pdf]

Table S2. Abstract reporting checklist required from the Preferred Reporting Items for Systematic Reviews and Meta-Analyses for Scoping Reviews (PRISMA-ScR) Checklist [15,16]

SECTION AND TOPIC	ITEM #	PRISMA-SCR CHECKLIST ITEM	Location where item is reported
**Title**			
Title	1	Identify the report as a scoping review.	Text on abstract
**Background**			
Objectives	2	Provide an explicit statement of the main objective(s) or question(s) the review addresses.	Text on abstract
**Methods**			
Eligibility criteria	3	Specify the inclusion and exclusion criteria for the review.	Text on abstract
Information sources	4	Specify the information sources (e.g. databases, registers) used to identify studies and the date when each was last searched.	Text on abstract
Risk of bias	5	Specify the methods used to assess risk of bias in the included studies.	NA
Synthesis of results	6	Specify the methods used to present and synthetize results.	Text on abstract
**Results**			
Included studies	7	Give the total number of included studies and participants and summarize relevant characteristics of studies.	Text on abstract
Synthesis of results	8	Present results for main outcomes, preferably indicating the number of included studies and participants for each. If meta-analysis was done, report the summary estimate and confidence/credible interval. If comparing groups, indicate the direction of the effect (i.e. which group is favored).	Text on abstract
**Discussion**			
Limitations of evidence	9	Provide a brief summary of the limitations of the evidence included in the review (e.g. study risk of bias, inconsistency and imprecision).	Text on abstract
Interpretation	10	Provide a general interpretation of the results and important implications.	Text on abstract
**Other**			
Funding	11	Specify the primary source of funding for the review.	NA
Registration	12	Provide the register name and registration number.	Text on abstract

NA: not applicable.
