# Supplementary material for: Predisposing Factors to Medication Errors by Nurses and Prevention Strategies: A Scoping Review of Recent Literature
Source: Nurs Rep. 2024 Jun 26;14(3):1553–69. doi: 10.3390/nursrep14030117 (PMC11270417; doi:10.3390/nursrep14030117)
Supplement: Supplementary file 1 [file nursrep-14-00117-s001.zip › Table_S3.pdf]

Table S3. Search strategy conducted in the PubMed, CINAHL (via EBSCO) and MEDLINE (via EBSCO) databases, on March 08, 2023.

SEARCH NO.	SEARCH TERMS AND EXPRESSIONS	RESULTS
	**PubMed**	
#1	“nurses” [Mesh] OR nurs * [TIAB]	559,604
#2	physicians [Mesh] OR students [Mesh] OR “nursing assistants” [Mesh] OR physician * [TIAB] OR student * [TIAB] OR “nursing assistant *” [TIAB] OR “nursing student *” [TIAB] OR “medical student *” [TIAB] OR undergraduate [TIAB] OR “nursing aide *” [TIAB] OR “nursing assistant *” [TIAB]	936,356
#3	#1 NOT #2	463,685
#4	“medical errors” [Mesh] OR “medication errors” [Mesh] OR “nursing error *” [TIAB] OR “medical error *” [TIAB] OR “medication error *” [TIAB] OR “medication administration error *” [TIAB] OR “medication preparation error *” [TIAB]	126,240
#5	“intensive care units” [Mesh] OR “respiratory care units” [Mesh] OR “coronary care units” [Mesh] OR “intensive medical care *” [TIAB] OR “intensive care *” [TIAB] OR ICU [TIAB] OR “care, intensive” [TIAB] OR “intensive care unit *” [TIAB] OR “intensive care medicine” [TIAB] OR “respiratory care unit *” [TIAB] OR “coronary care unit *” [TIAB]	250,749
#6	“intensive care units, pediatric” [Mesh] OR “intensive care units, neonatal” [Mesh] OR “intensive care units, pediatric” [TIAB] OR “intensive care units, neonatal” [TIAB]	27,043
#7	#5 NOT #6	223,706
#8	#3 AND #4 AND #7	370
#9	#3 AND #4 AND #7 from 2012–2023	**202**
	**CINAHL**	
S1	MH "Nurses" OR TI nurs* OR AB nurs*	713,823
S2	MH "Physicians” OR MH "Students+" OR MH "Nursing Assistants" OR TI physician* OR AB physician* OR TI student* OR AB student* OR TI "nursing assistant*" OR AB "nursing assistant*" OR TI “nursing student*” OR AB “nursing student*” OR TI “medical student*” OR AB “medical student*” OR TI undergraduate OR AB undergraduate OR TI “nursing aide*” OR AB “nursing aide*” OR TI “nursing assistant*” OR AB “nursing assistant*”	532,047
S3	S1 NOT S2	612,126
S4	MH "Treatment Errors" OR MH "Medication Errors" OR MH "Health Care Errors" OR TI "nursing error*" OR AB "nursing error*" OR TI “medical error*” OR AB “medical error*” OR TI “medication error*” OR AB “medication error*” OR TI “medication administration error*” OR AB “medication administration error*” OR TI “medication preparation error*” OR AB “medication preparation error*”	52,722
S5	MH "Intensive Care Units” OR MH "Respiratory Care Units" OR MH "Coronary Care Units" OR TI “intensive medical care*” OR AB “intensive medical care*” OR TI “intensive care*” OR AB “intensive care*” OR TI ICU OR AB ICU OR TI “care, intensive” OR AB “care, intensive” OR TI “intensive care unit*” OR AB “intensive care unit*” OR TI “intensive care medicine” OR AB “intensive care medicine” OR TI “respiratory care unit*” OR AB “respiratory care unit*” OR TI “coronary care unit*” OR AB “coronary care unit*”	121,377
S6	MH "Intensive Care Units, Pediatric" OR MH "Intensive Care Units, Neonatal" OR TI "intensive care units, pediatric" OR AB "intensive care units, pediatric" OR TI "intensive care units, neonatal" OR AB "intensive care units, neonatal"	22,666
S7	S5 NOT S6	98,711

S8	S3 AND S4 AND S7	337
S9	S3 AND S4 AND S7 from 2012–2023	**219**
	**MEDLINE**	
S1	MH "Nurses" OR TI nurs* OR AB nurs*	549,244
S2	MH "Physicians" OR MH "Students+" OR MH "Nursing Assistants" OR TI physician* OR AB physician* OR TI student* OR AB student* OR TI "nursing assistant*" OR AB "nursing assistant*" OR TI "nursing student*" OR AB "nursing student*" OR TI "medical student*" OR AB "medical student*" OR TI undergraduate OR AB undergraduate OR TI "nursing aide*" OR AB "nursing aide*" OR TI "nursing assistant*" OR AB "nursing assistant*"	943,938
S3	S1 NOT S2	454,611
S4	MH "Treatment Errors" OR MH "Medication Errors" OR MH "Health Care Errors" OR TI "nursing error*" OR AB "nursing error*" OR TI "medical error*" OR AB "medical error*" OR TI "medication error*" OR AB "medication error*" OR TI "medication administration error*" OR AB "medication administration error*" OR TI "medication preparation error*" OR AB "medication preparation error*"	27,163
S5	MH "Intensive Care Units" OR MH "Respiratory Care Units" OR MH "Coronary Care Units" OR TI "intensive medical care*" OR AB "intensive medical care*" OR TI "intensive care*" OR AB "intensive care*" OR TI ICU OR AB ICU OR TI "care, intensive" OR AB "care, intensive" OR TI "intensive care unit*" OR AB "intensive care unit*" OR TI "intensive care medicine" OR AB "intensive care medicine" OR TI "respiratory care unit*" OR AB "respiratory care unit*" OR TI "coronary care unit*" OR AB "coronary care unit*"	245,845
S6	MH "Intensive Care Units, Pediatric" OR MH "Intensive Care Units, Neonatal" OR TI "intensive care units, pediatric" OR AB "intensive care units, pediatric" OR TI "intensive care units, neonatal" OR AB "intensive care units, neonatal"	26,934
S7	S5 NOT S6	218,911
S8	S3 AND S4 AND S7	198
S9	S3 AND S4 AND S7 from 2012–2023	**126**
**TOTAL**		
