# Supplementary material for: Predisposing Factors to Medication Errors by Nurses and Prevention Strategies: A Scoping Review of Recent Literature
Source: Nurs Rep. 2024 Jun 26;14(3):1553–69. doi: 10.3390/nursrep14030117 (PMC11270417; doi:10.3390/nursrep14030117)
Supplement: Supplementary file 1 [file nursrep-14-00117-s001.zip › Table_S4.pdf]

Table S4. Overview of the characteristics of the studies included in the scoping review.

Author(s) (year), country	Source	Title	Study type	Participants	Aim(s)
Benoit et al. (2012), Switzerland	Acta Anaesthesiologica Scandinavica	Streamlining the medication process improves safety in the intensive care unit	Interrupted time-series	Nurses and nurses in an ICU	to evaluate the impact of a simplified process, of a new-designed medication order and nurses' documentation form, and of an education of the team to use it properly on medication errors.
Abbasinazari et al. (2012), Iran	Acta Medica Iranica	The Effect of Information Provision on Reduction of Errors in Intravenous Drug Preparation and Administration by Nurses in ICU and Surgical Wards	Observational descriptive study Educational method	Nurses from ICU and surgery wards	to evaluate the effect of nurses' education by a clinical pharmacist in reduction of errors in preparation and administration of IV drugs.
Breeding et al. (2013), Australia	Australian Critical Care	Medication Error Minimization Scheme (MEMS) in an adult tertiary Intensive Care Unit (ICU) 2009–2011	Plan-Do-Study-Act (PDSA) cycles associated with QI projects	46 nurses in the initial survey; 32 nurses in the follow-up survey	to improve medication safety in an adult intensive care setting (in a tertiary hospital) through the use/optimization of available resources.
Ferreira et al. (2014), Brazil	Revista de Enfermagem UFPE On Line	Knowledge of the nursing team about the rights of medication in Intensive Care Units	Descriptive and exploratory study with a quantitative approach	29 nurses from the Intensive Care Unit of a university hospital	to identify the knowledge of the nursing staff of the intensive care units of a university hospital about the rights applied in the medication process.
Durham et al. (2016), USA	The Journal of Nursing Administration	Reducing medication administration errors in acute and critical care: multifaceted pilot program targeting RN awareness and behaviors	Observational time-series	NA	To increase the nurse's sensitivity to the potential risk of error, improve behaviors and reduce observed medication administration errors.
Tan et al. (2017), Malaysia	Journal of Pharmacy Practice and Research	The effect of education intervention on parenteral medication preparation and administration among nurses in a general intensive care unit	Direct observational study using pre- and post-intervention measurements	39 nurses in pre-intervention; 35 nurses in post-intervention	To measure the effects of education intervention on the incidence and types of parenteral medication preparation and administration errors, adherence to good practices, and the accuracy of drug infusions' concentrations.
Di Muzio et al. (2017), Italy	Applied Nursing Research	Knowledge, behaviours, training and attitudes of nurses during preparation and administration of intravenous medications in intensive care units (ICU). A multicenter Italian study	Cross-sectional study (multicentre)	529 nurses who work in the ICUs of Southern, Centre and Northern Italy hospitals	To describe the knowledge, attitudes, behaviours and training needs of the Italian nurses who work in ICUs towards the use of IV drugs, and to identify the strategies that nurses can adopt to prevent the occurrence of medication errors.
Xu et al., (2017), USA	Nursing Research	Facilitated Nurse Medication-Related Event Reporting to Improve Medication Management Quality and Safety in Intensive Care Units	Observational study	109 nurses in three different ICUs were observed in 153 situations	to explore the utility of facilitated medication-related events reporting in identifying system deficiencies, and the relationship between especially medication-related events and nurses' work in the ICUs.

Santomauro et al. (2018), Australia	Journal of Patient Safety	Interruptions to Intensive Care Nurses and Clinical Errors and Procedural Failures: A Controlled Study of Causal Connection	Prospective controlled trial	70 ICU nurses	to investigate whether ICU nurses who receive a higher number of workplace interruptions commit more clinical errors and procedural failures than those who receive a lower number of interruptions.
Adebayo (2018), USA	Journal of Informatics Nursing	Strategies for Improving Documentation of Medication Overrides	Plan Do Study Act (PDSA) technique was used to guide a pilot study. Continuous improvement project.	Nurses from Medical ICU and Surgical ICU (all nurses on weekday, weekend, day and night shifts in both units)	to educate nurses on how to link override medications to orders to reduce administration errors.
Gracia et al. (2019), Spain	BMC Health Services Research	Medication errors and drug knowledge gaps among critical-care nurses: a mixed multi-method study	Mixed study (multi-method)	ICU nurses	To identify the main medication errors that occur in the ICU at a general hospital.<br>To study if the level of knowledge that critical-care nurses have about the use and administration of medications is related to the most common medication errors.
Eltaybani et al. (2020), Egypt	Journal of Nursing Management	Recommendations to prevent nursing errors: Content analysis of semi-structured interviews with intensive care unit nurses in a developing country	Cross-sectional narrative qualitative	108 ICU nurses	To elicit intensive care unit nurses' recommendations to prevent nursing errors.
Suclupe et al. (2020), Spain	Journal of Advanced Nursing	Medication errors in prescription and administration in critically ill patients	Observational, analytical and cross-sectional	Intensive care unit and intermediate care unit nurses	To determine the prevalence and magnitude of medication errors and their association with patients' socio-demographic and clinical characteristics and nurses' work conditions.
Yu et al. (2020), China	Drugs & Therapy Perspectives	Reduce medication errors in tube feeding administration by establishing administration standards and standardizing operation procedures	Retrospective and observational	ICU nurses	To evaluate and to reduce the prevalence of medication errors associated with the tube feeding administration through the development and implementation of a standardised operating procedure.
Gracia et al. (2020), Spain	Journal of Advanced Nursing	Medication errors and risk areas in a critical care unit	Descriptive, longitudinal and retrospective	30 ICU nurses	To identify the main medication errors, their causality and the highest risk areas in critical care.
Ek et al. (2022), Norway	Nordic Journal of Nursing Research	ICU nurses' experiences of medication double-checking: A qualitative study	Qualitative and explorative	5 ICU nurses	To explore intensive care nurses' experiences and perceptions of dual medication control.

NA: not applicable.
